# Long-term dynamics and driving mechanisms of plant communities in a temperate estuary in eastern China based on pollen analysis: a case study of the Liaohe Estuary

**DOI:** 10.3389/fpls.2025.1578390

**Published:** 2025-04-28

**Authors:** Haoran Liu, Weiwei Liu, Jinzhi Wang, Wei Li, Jingwen Zhang, Jian Gong, Lijuan Cui

**Affiliations:** ^1^ Key Laboratory of Wetland Conservation and Restoration, Chinese Academy of Forestry, Beijing, China; ^2^ Institute of Wetland Research, Chinese Academy of Forestry, Beijing, China; ^3^ Institute of Ecological Protection and Restoration Research, Chinese Academy of Forestry, Beijing, China

**Keywords:** plant community characteristics, estuary, pollen analysis, environmental factors, human activities

## Abstract

**Introduction:**

The Liaohe Estuary, a representative estuarine ecosystem in eastern China, has experienced significant shifts in plant community characteristics due to climate change and anthropogenic influences in recent decades.

**Methods:**

This study employed sediment 210Pb dating, pollen analysis, and environmental factor indicators to comprehensively assess the composition, trends, and drivers of plant communities in the Liaohe Estuary from 1944 to 2022.

**Results:**

The findings revealed that herbaceous plants dominated the estuary's vegetation under a cool and humid climate, though humidity exhibited a declining trend over time. Between 2001 and 2022, pollen concentration and herbaceous plant prevalence increased significantly. Key environmental drivers—mean annual temperature (MAT), salinity, grain size, pH, and agricultural production—were strongly correlated (p < 0.001) with plant community dynamics. Natural factors (grain size, salinity) enhanced the dominance of key species but reduced overall pollen concentration. Conversely, agricultural activities diminished dominant species proportions while increasing pollen concentration.

**Discussion:**

These results highlight the dual influence of climatic and anthropogenic factors on estuarine vegetation. The study provides a theoretical basis for restoring degraded estuarine ecosystems.

## 1 Introduction

The Liaohe Estuary, a vital coastal ecosystem in northern China, is one of the most representative estuary in high-latitude regions (Jiang et al., 2015). Its unique geographical environment and complex ecological processes play a crucial role in preserving regional biodiversity and maintaining ecological balance. Furthermore, it serves as a valuable indicator of the impacts of climate change and land-use changes driven by human activities (Xiong et al., 2023). However, estuarine ecosystems are among the most fragile ecosystems globally, highly sensitive to external disturbances and vulnerable to the combined effects of from physical, chemical, and biological processes. In recent years, global climate change and human activities have led to a 14.8% reduction in the Liaohe Estuary area, accelerating the succession of plant communities. These changes have significantly altered the structure and function of plant communities in the area, posing serious threats to biodiversity conservation and the preservation of wildlife habitats (Xie et al., 2018; Peng et al., 2021; Cui et al., 2022). Therefore, it is necessary to study the succession characteristics of plant communities in the Liaohe Estuary to support its sustainable development.

Estuarine plant communities play a vital role in supporting biodiversity. Exploring their characteristics is of significant value for understanding estuarine ecosystem functions, maintaining biodiversity, and responding to environmental changes (Zuo et al., 2012). Research on estuarine plant communities primarily utilizes modern vegetation diversity indicators, such as species richness, evenness, and coverage, to analyze the response factors of plant communities and study plant community succession based on long-term trends in plant diversity. Radiometric dating can provide precise chronological sequences, offering a high-resolution temporal framework for studying long-term trends in plant community changes (Appleby and Oldfield, 1978). However, there is a notable lack of comprehensive studies examining the characteristics, changes, and driving factors of plant communities over long-term scales using radiometric dating (Sun et al., 2020; Lane et al., 2024). Estuarine plant communities are constrained by various environmental factors, with climate change—particularly fluctuations in temperature and precipitation—significantly influencing their structure and distribution. Changes in precipitation, for example, alter freshwater flow, indirectly impacting soil salinity in estuary (Lorenz et al., 2021; Feng and Chen, 2023; Byrne et al., 2023). This, in turn, affects species composition, as well as the speed and direction of plant community succession (Byrne et al., 2001; Duarte et al., 2013). Human activities, such as agricultural production, directly damage estuarine plants and soils, leading to changes in plant species composition (Zheng et al., 2004; Deng et al., 2006; Abrahim et al., 2013). Furthermore, abiotic factors such as salinity, pH, and grain size are widely recognized as key determinants of the distribution patterns of dominant plants in estuaries (Fenu et al., 2012; Meixler et al., 2018; Pratolongo et al., 2019; Borde et al., 2020). Therefore, changes in both the natural environment and human activities have impacts on plant communities. In exploring the succession of plant communities, it is essential to thoroughly analyze the combined effects of environmental factors and human activities on the structure and function of plant communities. This will help to comprehensively understand the driving mechanisms of plant community succession.

Pollen analysis, as a valuable tool for investigating historical ecological characteristics, offers critical insights into past vegetation composition, coverage, and environmental changes. It plays a key role in understanding historical dynamics of plant communities (Xiao et al., 2008; Wang, 2009; Li and Zhao, 2018). However, the application of paleoenvironmental methods, such as pollen analysis and ^210^Pb dating, to modern ecological research on estuarine systems, particularly in the unique context of the Liaohe Estuary, has not been previously explored. In this study, a sediment core was collected from the tidal flats of the Liaohe Estuary, pollen analysis and ^210^Pb dating were systematically integrated to establish a chronological framework for plant communities in this region. Based on pollen assemblages and concentrations, the composition and trends of plant communities in the Liaohe Estuary were comprehensively analyzed. By incorporating multiple environmental indicators, including mean annual temperature (MAT), mean annual precipitation (MAP), soil salinity, grain size and agricultural production, this study not only elucidated the combined impacts of climate change and human activities on plant community changes but also provided a scientific basis for vegetation restoration and ecological reconstruction of degraded estuarine ecosystems. This research fills a critical gap in the study of plant community dynamics in the Liaohe Estuary, offering significant insights into the evolution and driving mechanisms of its ecosystem over recent decades, as well as practical guidance for sustainable development strategies in the region.

## 2 Materials and methods

### 2.1 Sampling location and core collection

The Liaohe Estuary (40°45′—41°05′ N, 121°28′—121°58′ E) is located at the mouth of the Liaohe River in Panjin, Liaoning Province, China. It is the largest reed coastal wetland reserve in China, as well as the second-largest reed-producing area in the world, covering approximately 2.23×10^5^ hm² (Ma et al., 2017). The region has a temperate semi-humid monsoon climate with distinct seasons and concurrent heat and rain. The average annual temperature is 8.3 °C–8.4 °C, with the lowest temperature in January reaching –29.2 °C and the highest temperature in July reaching 35.2 °C. The frost-free period lasts an average of 171 days, and the average annual precipitation is 631 mm, mainly in the period from July to September. The dominant species in the study area are *Spartina alterniflora*, *Scirpus triqueter*, and *Suaeda salsa*.

**Figure 1:**
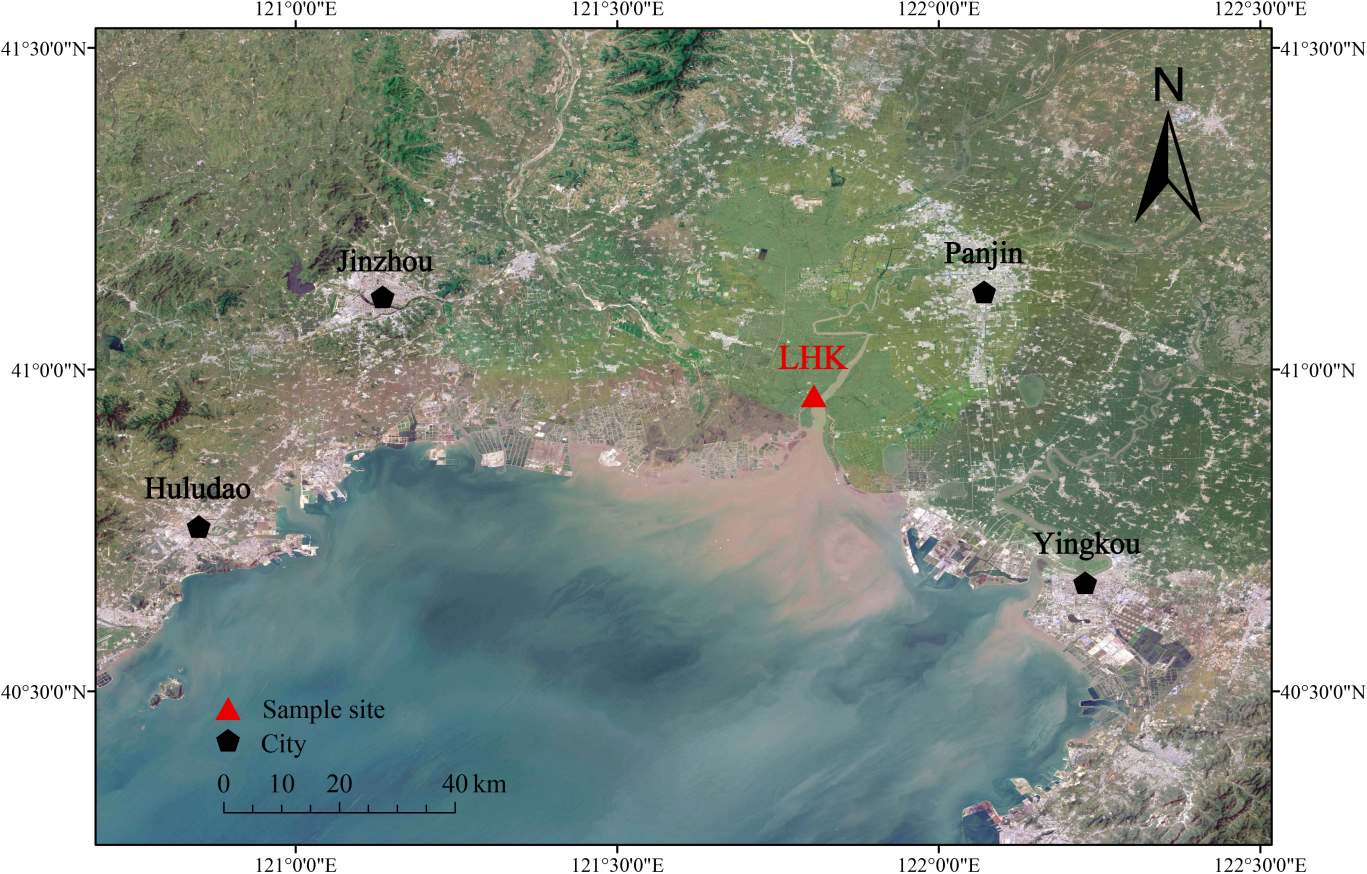
Location map of core LHK in Liaohe Estuary.

Core Liaohekou (LHK, 40°57′26″ N, 121°48′00″ E) was collected from the Liaohe estuary National Nature Reserve in Panjin, Liaoning Province, in May 2023. The core has a total length of 154 cm (**Figure 1**). The LHK site was located within the intertidal mudflat dominated by *S. alterniflora* and *S. triqueter*. Samples were collected using an electroacoustic sediment sampler (diameter: 90 mm). Immediately after collection, the samples were sectioned into 2 cm intervals, preserved in an incubator, and stored at 4 °C in the refrigerator upon transport to the laboratory. A total of 77 samples were collected.

### 2.2 Chronometry

The ^210^Pb dating method is a technique used to determine the age of recent sediments based on the radioactive decay properties of ^210^Pb (Goldberg, 1963). The chronological framework of the sediment core soil profile was established through ^210^Pb dating analysis. For the 77 sediment samples obtained, 7–8 g of each soil sample was freeze-dried. Subsequently, ^210^Pb specific activity was measured using a gamma spectrometry system comprising a high-purity germanium well detector (Ortec HPGe GWL-120-15) manufactured by EG & G Ortec, an Ortec-919 quadruple spectrometer controller, and an IBM computer with a 16 K multichannel analyzer, ^210^Pb activity was measured at 46.5 keV. Once radioactive equilibrium was achieved, the isotope ^214^Pb of ^210^Pb after reaching radiation equilibrium was measured at 352 keV using gamma rays. Standard ^210^Pb samples provided by the University of Liverpool were used for calibration, with a measurement error of less than 10%.

### 2.3 Pollen analysis

Pollen analysis is a technique first proposed by von Post in 1916, which reconstructs paleovegetation, paleoclimate, and paleoenvironments by studying pollen and spores in sediments (von Post, 1916). The 77 sediment samples were consolidated by combining every two consecutive layers, resulting in a total of 39 sediment samples (with the final layer, 152–154 cm, treated as a single sample). Approximately 5 g of fresh soil was collected from each sample for pollen concentration measurement and identification. For each sample, at least 300 pollen grains were analyzed, with an average of 366 grains per sample. The identification process relies on key reference materials, including the pollen images and morphological descriptions in books such as *Pollen Morphology of Chinese Plants* (Wang et al., 1995). Modern pollen databases from Sun Yat-sen University and international cooperative laboratories were also utilized, supplemented by slide-based microscopic morphological comparison analyses. Pollen analysis of the sediment profile was performed using CONISS cluster analysis, dividing the profile into four distinct pollen zones. This classification was based on the sum of squared differences and similarity indices calculated between samples.

Pollen concentration represents the number of pollen per unit volume or sample weight (Nalepka and Walanus, 2003). It is calculated by counting the total number of pollen in the sample and measuring the sample weight. The formula is as follows:


$$X=\frac{N_{n}}{N^{\prime }G}$$

where: X represents the pollen concentration (grains/g); N represents the number of marker pollen added to the sample; N’ represents the number of marker pollen counted under the microscope; n represents the total number of pollen counted under the microscope; G represents the weight of the sample used for analysis.

### 2.4 Determination of soil physicochemical properties

Similar to the pollen analysis, 39 sediment samples were collected for measurements of soil grain size, salinity, and pH. Soil grain size was determined using laser diffraction technology. Organic matter was removed using H_2_O_2_, carbonates were eliminated with 10% hydrochloric acid, and dispersion was achieved with sodium hexametaphosphate (NaPO_3_)_6_. Soil grain size was then determined using a Mastersize 2000 laser particle size analyzer (Malvern Instruments, UK). Soil salinity was measured using the conductivity method, with a DJS-1C conductivity meter, and conductivity of the DDSJ-30–8 sediment extract was calculated (Ryan et al., 1996). Soil pH was determined using the pH electrode method, where a pH meter was used to measure the pH of the extract from a soil-water mixture (ratio of 5:1) obtained from the sediment samples.

### 2.5 Climate and agricultural production data

Based on the 1 km resolution monthly temperature and precipitation dataset from the National Tibetan Plateau Scientific Data Center, the mean annual temperature (MAT) and mean annual precipitation (MAP) for Panjin City, Liaoning Province, from 1944 to 2022 were calculated, yielding the MAT and MAP data (Peng, 2019, 2020). The agricultural production data for Panjin City, Liaoning Province, are numerical data. The data from 1990 to 2018 were obtained from the National Bureau of Statistics, while data from 2019 to 2022 were retrieved from the *Panjin Statistical Yearbook*. Missing agricultural production data for the period of 1944 to 1989 were estimated using the agricultural production growth rate provided in the *Panjin Statistical Yearbook*.

### 2.6 Data analysis

Prior to statistical analysis, all raw data were organized and preprocessed using Microsoft Excel 2019 (Held et al., 2019). Tilia software was used to explore the changes in pollen percentages at different soil depths, while Origin 2021 was employed to investigate the variations in the percentages of pollen taxa and pollen concentration with soil depth (Grimm, 1990; Moberly et al., 2018). Principal component analysis (PCA) of the most dominant seven pollen taxa was conducted using Origin 2021 to explore the distribution characteristics of the most dominant seven pollen taxa. Redundancy analysis (RDA) of the most dominant seven pollen taxa, pollen concentration, and environmental factors was performed using R 4.3.3 to examine the influence of environmental factors on the most dominant seven pollen taxa and pollen concentration (R Core Team, 2024). The rdacca.hp package was utilized to extract the explanatory power of each environmental factor, aiming to assess the contribution of each environmental factor to the most dominant seven pollen taxa and pollen concentration (Cheng et al., 2023). Additionally, based on the random forest model, R 4.3.3 was used to analyze the contribution of principal components of the most dominant seven pollen taxa, pollen concentration, and environmental factors, which was cross-validated with the RDA results to confirm the contributions of environmental factors to the most dominant seven pollen taxa and pollen concentration. Mapping was accomplished using Tilia, Origin 2021, R 4.3.3, and ArcMap 10.7 (Johnston et al., 2001).

## 3 Results

### 3.1 Pollen composition characteristics

**Figure 2:**
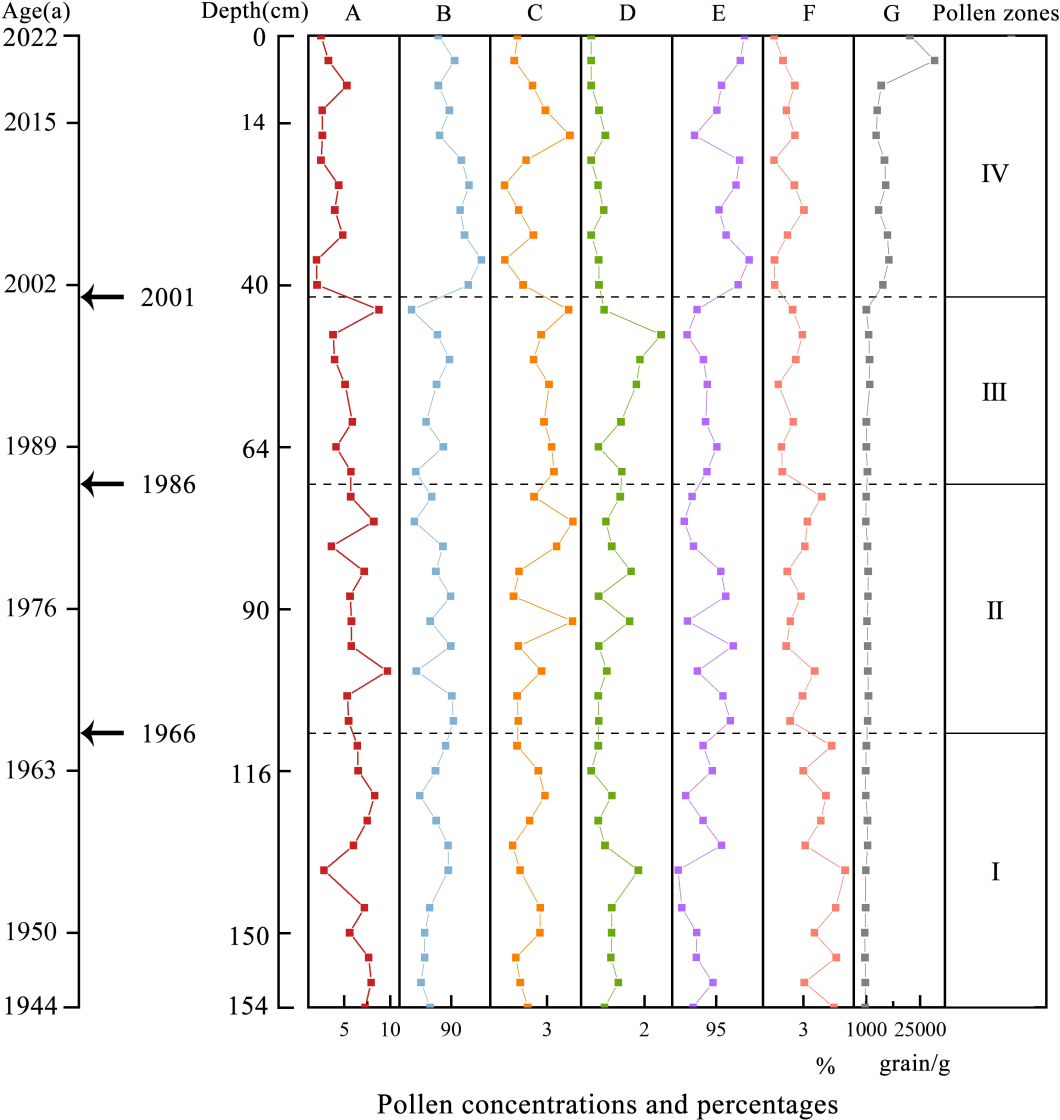
Integrated column of pollen concentrations and percentages from core LHK in Liaohe Estuary Wetland. **(A)** Woody pollen; **(B)** Herbaceous pollen; **(C)** Fern spores; **(D)** Algae spores; **(E)** Terestrial pollen; **(F)** Aquatic pollen; **(G)** Pollen concentration.

**Figure 3:**
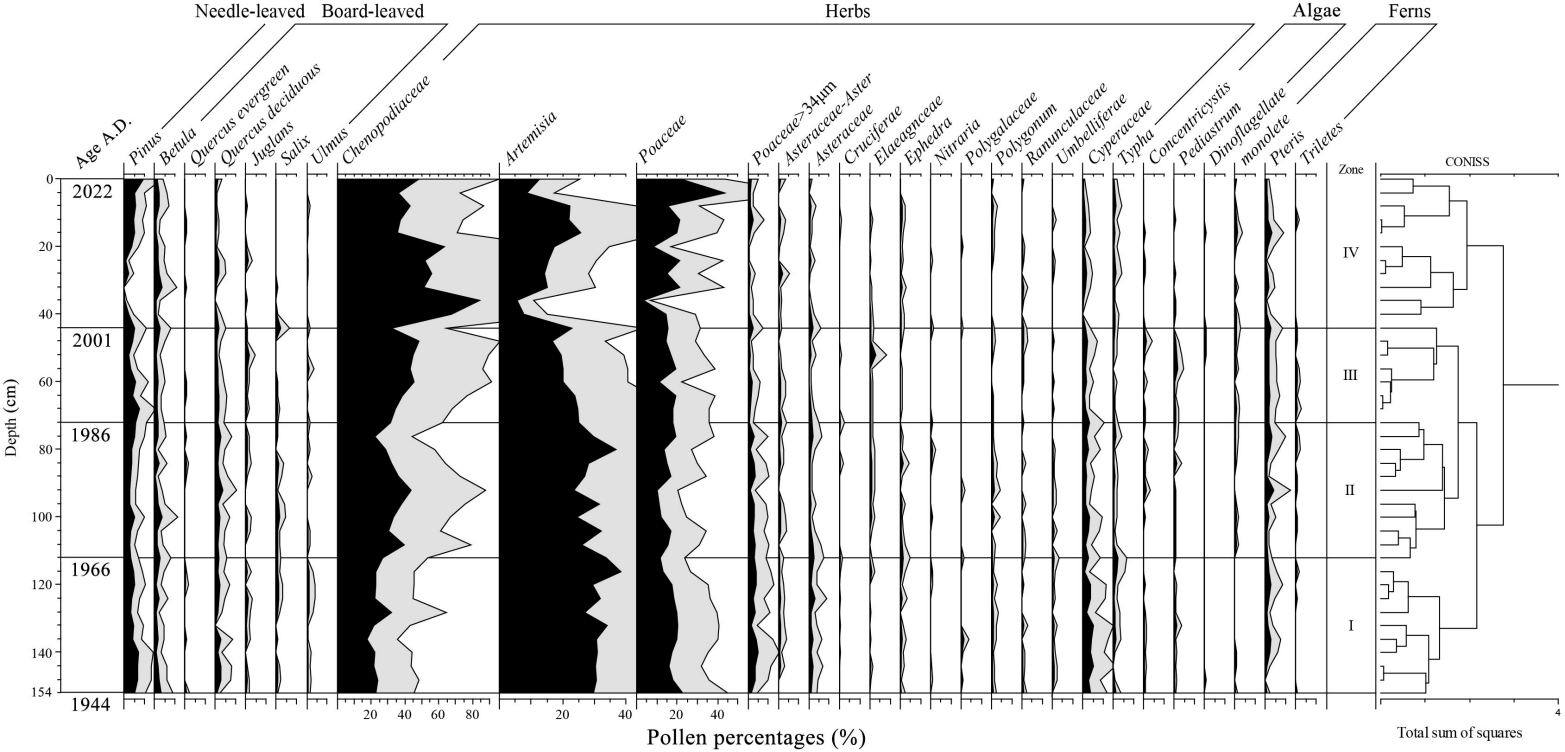
Variations of the pollen assemblages (%) along the core LHK.

Since it is challenging to identify some pollen from the family to the genus level (e.g., *Poaceae*), certain pollen grains were identified to the genus level (e.g., *Pinus*), while others were identified to the family level. A total of 14289 pollen grains were identified from core LHK, belonging to 68 categories at the family and genus levels. The sediment profile contained the following pollen taxa: 25 taxa of herbaceous pollen, 33 taxa of woody pollen, 7 taxa of fern spores, and 3 taxa of algal spores. Among these, three aquatic pollen taxa of were identified: *Cyperaceae*, *Typha*, and *Myriophyllum*, while the remaining 65 types were terrestrial. Herbaceous pollen was predominantly represented by *Artemisia*, *Asteraceae-Aster*, *Asteraceae*, and *Poaceae*, followed by *Cyperaceae* and *Typha*, with occasional occurrences of *Elaeagnaceae*, *Ephedra*, *Nitraria*, *Polygalaceae*, *Polygonum*, *Ranunculaceae*, and *Umbelliferae*. Woody pollen mainly included *Pinus*, *Betula*, deciduous *Quercus*, *Salix*, *Ulmus*, and evergreen *Quercus*. Fern spores were primarily represented by *Pteris*, as well as monolete and trilete spores. Algal spores were relatively sparse, including *Concentricystis*, *Pediastrum*, and *Dinoflagellate*. Based on the average percentage of each pollen taxan in the entire sediment profile, herbaceous pollen dominated with 87.6% of the total pollen, followed by woody pollen (9.6%), fern spores (2.1%), and algae spores (0.7%), with the latter representing a relatively small proportion (**Figures 2**, **3**).

### 3.2 Pollen zone characteristics and differences among pollen zones

The differences in pollen composition between adjacent samples were compared for all identified herbaceous and woody pollen, fern and algal spores, and the results were divided into four pollen zones, each characterized by unique pollen assemblage characteristics. In addition, an analysis was conducted based on the percentage of pollen taxa in each pollen zone (**Figure 3**).

Zone I: *Artemisia*-*Betula*-*Poaceae*-*Pinus* (112–156 cm)

The total pollen concentration was low, with an average of 883 grains g^-1^ (**Figure 2**). The pollen was mainly herbaceous pollen (82.9%–89.3%, average 85.9%), followed by woody pollen (7.6%–14.6%, average 11.7%). Fern spores (1.07%–2.86%, average 1.74%) and algae spores (0%–1.8%, average 0.7%) had lower pollen shares (**Figure 2**). Herbaceous pollen was dominated by *Artemisia* (32.2%), *Chenopodiaceae* (23.9%), and *Poaceae* (21.6%), with other terrestrial pollen such as cold-wet-loving *Asteraceae* (1.7%) and aquatic pollen such as *Cyperaceae* (3.6%) and *Typha* (1.0%). The most common woody pollen was *Pinus* (5.2%), followed by *Betula* (1.8%), deciduous *Quercus* (1.7%), *Ulmus* (0.7%), *Salix* (0.5%), and *Juglans*. Fern spores mainly included the moisture-loving *Pteris* (1.4%), as well as trilete and *monolete* spores, while algae spores included *Pediastrum* and *Concentricystis*.

Zone II: *Chenopodiaceae*-*Artemisia*-*Poaceae* (72–112 cm)

The total pollen concentration was clearly higher than that of the Zone I, with an average concentration of 1567 grains g^-1^ (**Figure 2**). It was still dominated by herbaceous pollen (81.6%–90.5%, average 87.0%), followed by woody pollen (7.5%–13.2%, average 10.0%), with sporadic occurrences of fern spores (0.9%–4.7%, average 2.3%) and algae spores (0.3%–1.5%, average 0.7%) in this zone (**Figure 2**). Among the herbaceous pollen, compared to Zone I, *Chenopodiaceae* (34.6%) showed a significant increase in proportion, whereas the proportions of *Artemisia* (29.2%) and *Poaceae* (18.0%) slightly decreased, and *Cyperaceae* (2.2%), *Asteraceae* (1.2%), and *Typha* (0.7%) were present. The proportions of aquatic pollen, such as *Cyperaceae* and *Typha*, decreased comparing to Zone I. The woody pollen was still dominated by *Pinus* (3.9%), followed by deciduous *Quercus* (2.0%), *Betula* (1.7%), *Salix* (0.7%), and *Juglans* (0.4%). Fern spores were still mainly *Pteris* (1.7%), followed by *monolete* and *trilete* spores. Algae spores included small concentrations of *Pediastrum* and *Concentricystis*.

Zone III: *Chenopodiaceae*-*Artemisia*-*Poaceae*-*Pinus* (44–72 cm)

The pollen concentration was similar to that of the Zone II, at 1738 grains g^-1^ (**Figure 2**). This zone was again dominated by herbaceous pollen (81.0%–89.6%, average 85.5%), followed by woody pollen (6.4%–14.1%, average 10.1%). Fern spores (2.1%–4.4%, average 3.1%) and algae spores (0.3%–2.6%, average 1.3%) were still scarce (**Figure 2**). Among the herbaceous pollen, compared to Zone II, *Chenopodiaceae* (42.8%) increased and became the most dominant, followed by *Artemisia* (22.1%) and *Poaceae* (18.6%), with lower abundances of *Cyperaceae* (1.6%), *Asteraceae* (0.9%), and *Asteraceae-Aster* (0.6%), etc. While the proportion of aquatic pollen decreased further compared with Zone II. The woody pollen was dominated by *Pinus* (4.9%), followed by *Betula* (1.6%), deciduous *Quercus* (1.4%), Sporadic *Juglans* (0.6%), and *Salix* (0.5%). Fern spores mainly included *Pteris* (2.1%) and monoletes (0.6%), and algae spores mainly included *Pediastrum* (0.8%), with an increased pollen percentage compared to Zone II.

Zone IV: *Chenopodiaceae*-*Poaceae*-*Artemisia* (0–44 cm)

The pollen concentration clearly increased compared to that in the Zone III, being the highest in the entire profile at 11504 grains g^-1^ (**Figure 2**). The zone was dominated by herbaceous pollen (87.1%–94.0%, average 91.3%), relative to Zone III, with a significant increase in percentage, accounting for the highest proportion in the entire profile (**Figure 2**). We also observed a significant decrease in woody pollen (2.6%–11.8%, average 6.9%), which increased with Zone III. Fern spores (0.3%–4.5%, average 1.7%) and algae spores (0%–0.5%, average 0.2%) were clearly decreased compared to Zone III. Among the herbaceous pollen, *Chenopodiaceae* (53.1%) continued to increase compared to Pollen Zone III, dominating the assemblage, followed by *Poaceae* (20.2%), while *Artemisia* (15.4%) clearly decreased. *Cyperaceae* (0.9%) and *Typha* (0.8%) were also present. Woody pollen was dominated by *Pinus* (3.7%), followed by *Betula* (1.7%), while deciduous *Quercus* (0.7%) was the main deciduous pollen with decreased percentage compared to Zone III. Fern spores mainly included *Pteris* (1.6%), with a significant decrease in proportion compared with Zone III, whereas algae spores were almost absent.

The percentage of *Chenopodiaceae* in the Liaohe Estuary varied clearly across different pollen zones, increasing by 29.2% from pollen zone I to pollen zone IV, while the percentage of *Artemisia* decreased by 16.8%. Specifically, *Artemisia* was most prevalent in pollen zone I, whereas *Chenopodiaceae* became dominant in pollen zones II to IV. According to the ^210^Pb dating analysis, core LHK showed sedimentation spanning from 1944 to 2022. Between 2001 and 2022, pollen concentration increased clearly (by a factor of 6.7 compared to the previous pollen zone), with the percentage of herbaceous pollen increasing by 6.8%, while the percentages of woody pollen, fern spores, and algae spores decreased. Additionally, over time, the percentage of terrestrial pollen increased by 3.4%, while that of aquatic pollen decreased (**Figure 2**).

### 3.3 PCA of main the most dominant seven pollen taxa

**Figure 4:**
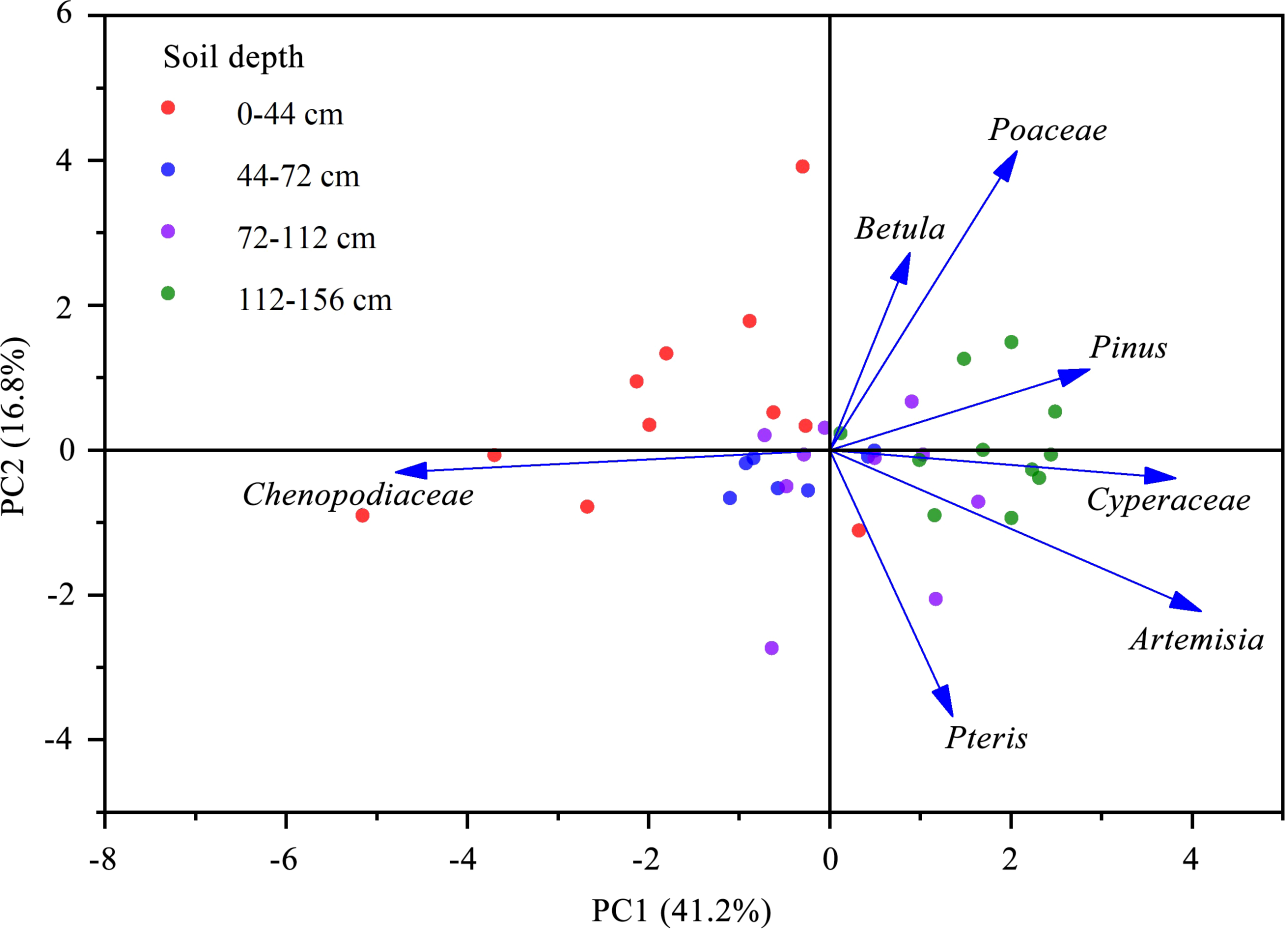
Principal component analysis of the most dominant seven pollen taxa.

Among the pollen taxa shown in **Figure 3**, the most dominant seven pollen taxa, namely *Pinus*, *Betula*, *Chenopodiaceae*, *Artemisia*, *Poaceae*, *Cyperaceae*, and *Pteris*, each account for more than 1.5% of the total pollen in each pollen zone. PCA was conducted on the most dominant seven pollen taxa. The results showed that the first two axes could explain 58.0% of the variations in main pollen percentage, with the first principal component accounting for 41.2%, which was noticeably higher than the second principal component of 16.8% (**Figure 4**). On the first principal component axis, *Betula*, *Pinus*, *Poaceae*, *Cyperaceae*, *Artemisia*, and *Pteris* showed a positive correlation, whereas *Chenopodiaceae* showed a negative correlation. This indicates that except for *Chenopodiaceae*, the other main pollen taxa have similar characteristics.

### 3.4 Correlation analysis of pollen and environmental factors

**Figure 5:**
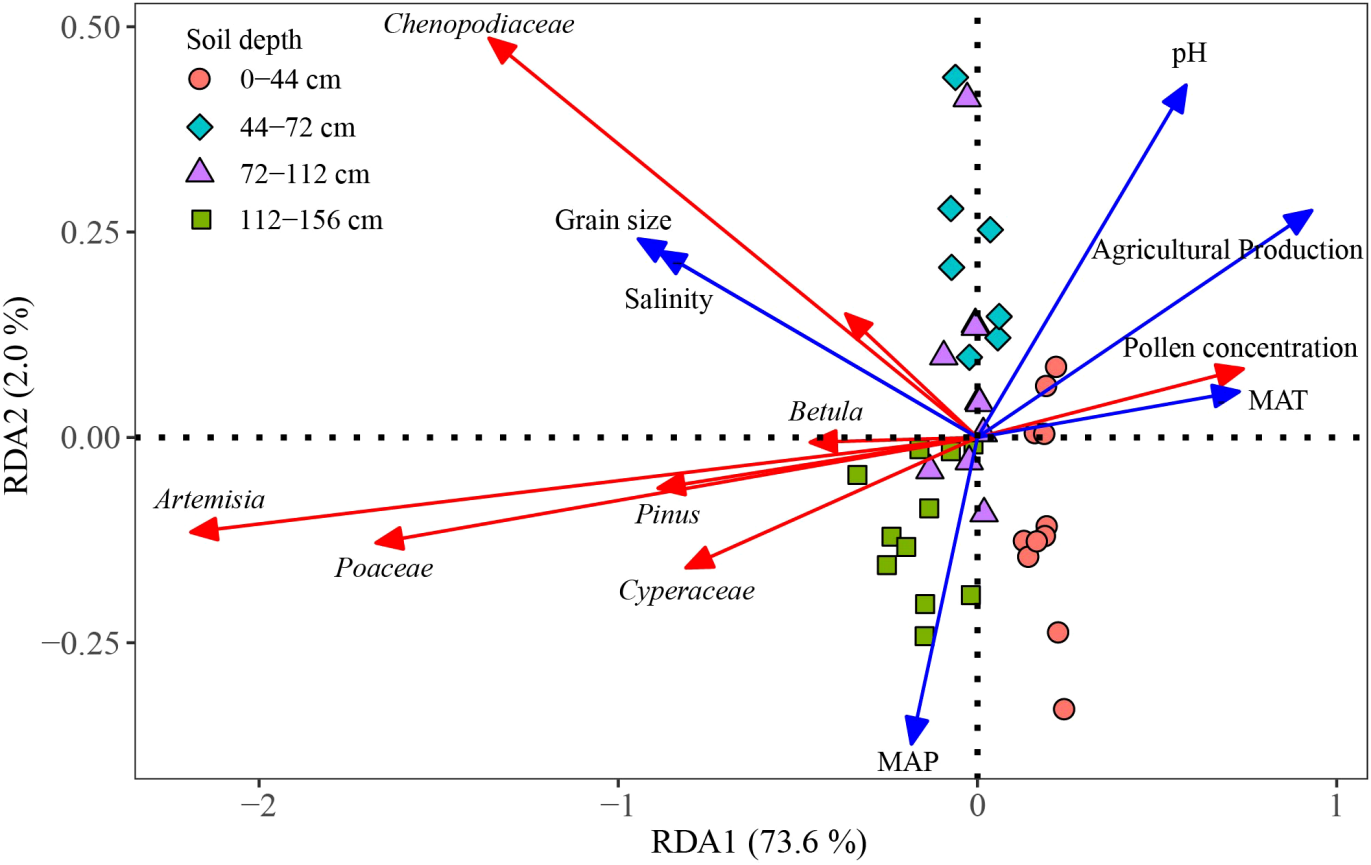
Redundancy analysis of the most dominant seven pollen taxa and environmental variables. MAT, Mean Annual Temperature; MAP, Mean Annual Precipitation.

Between 2001 and 2022, with the increase in MAT (Mean Annual Temperature), the percentages and concentrations of herbaceous pollen significantly increased, while those of woody plants, ferns, and algae showed a notable decrease (**Supplementary Figure S1**). To further analyze the relationship between environmental factors and the most dominant seven pollen taxa as well as pollen concentration, climate factors (MAT and MAP), soil factors (salinity, grain size, and pH), and human activity factors (agricultural production) were selected for correlation analysis with the percentages of the most dominant seven pollen taxa and pollen concentration (**Figure 5**). Through Detrended Correspondence Analysis (DCA), the sum of the first four axis eigenvalues was 0.19 (< 3), indicating that a linear model RDA was appropriate to explain the impact of environmental factors on the most dominant seven pollen taxa and pollen concentration. The results showed that the environmental factors explained 67.4% of the variation in the community of the most dominant seven pollen taxa, with the first axis explaining 73.6% and the second axis explaining 2.0%. Grain size (30.8%, *p*< 0.001), agricultural production (29.7%, *p*< 0.001), MAT (11.4%, *p*< 0.001), salinity (21.2%, *p<* 0.001) and pH (6.9%, *p<* 0.001) significantly explained these differences (**Table 1**). In the different sediment layers, MAT and agricultural production had a notable impact on the 0–44 cm sediment layer.

**Table 1:** Results of redundancy analysis interpreting the percentage of the most dominant seven pollen taxa by environmental factors.

Environmental factor	Percentage of interpretations (%)	P-value
MAT (Mean Annual Temperature)	11.4	<0.001
MAP (Mean Annual Precipitation)	0	>0.05
Salinity	21.2	<0.001
pH	6.9	<0.001
Grain size	30.8	<0.001
Agricultural production	29.7	<0.001

**Figure 6:**
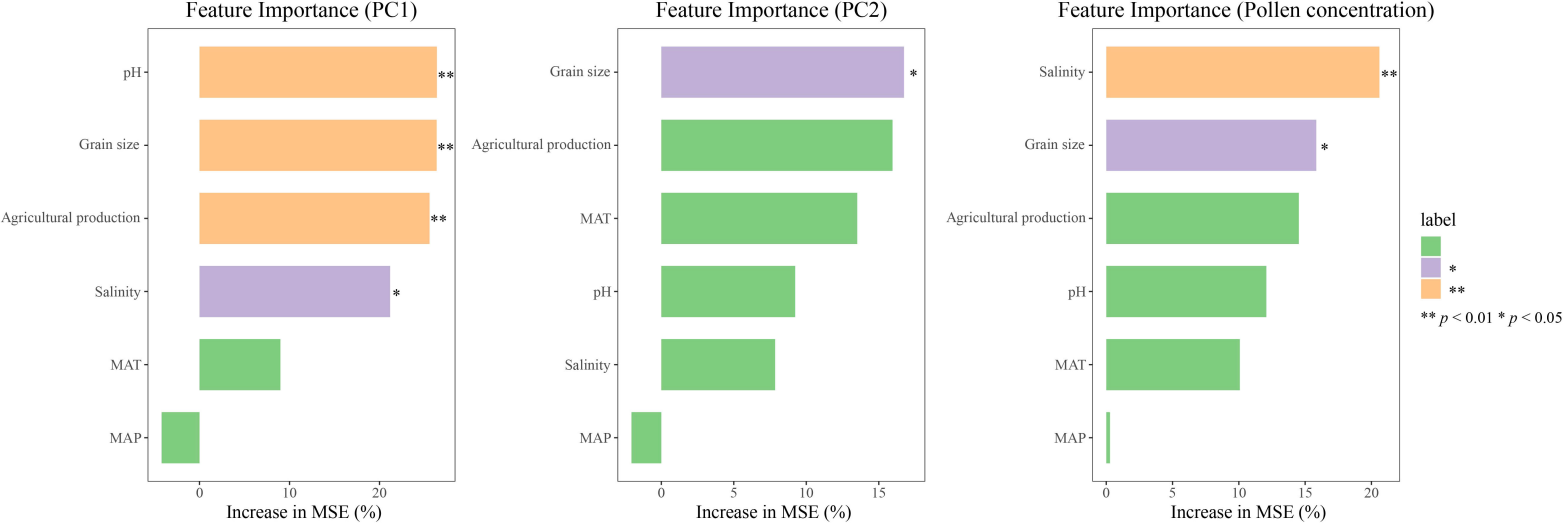
Contribution analysis of environmental factors to the principal components of the seven most dominant pollen taxa and pollen concentration based on random forest. MAT, Mean Annual Temperature; MAP, Mean Annual Precipitation. Significance levels: *p < 0.05, **p < 0.01.

Based on the aforementioned PCA results, the first two principal components (PC1 and PC2) of the most dominant seven pollen taxa were extracted. Combined with pollen concentration, the random forest model was used to explore the contribution of environmental factors to the principal components of the most dominant seven pollen taxa and pollen concentration. The results showed that pH, salinity, Grain size, Agricultural production, and MAT ranked at the top in terms of their contributions to PC1, PC2, and pollen concentration, while MAP had a lower contribution (**Figure 6**). pH, Grain size, and Agricultural production had extremely significant contributions to PC1 (*p*< 0.01), and salinity had a significant contribution to PC1 (*p*< 0.05). The contribution ranking was pH > Grain size > Agricultural production > Salinity > MAT > MAP. Grain size had a significant contribution to PC2 (*p*< 0.05). The contribution ranking was Grain size > Agricultural production > MAT > pH > Salinity > MAP. Salinity had an extremely significant contribution to pollen concentration (*p*< 0.01), and Grain size had a significant contribution to pollen concentration (*p*< 0.05). The contribution ranking was Salinity > Grain size > Agricultural production > pH > MAT > MAP.

## 4 Discussion

### 4.1 Characteristics of pollen of plant communities and climate change in the Liaohe Estuary

The entire sediment core profile showed that pollen concentration increased with decreasing soil depth, which was particularly evident in pollen zones III and IV (**Figure 2**). Pollen concentration can effectively reflect changes in pollen productivity, and studies also have shown a significant positive correlation between pollen concentration and vegetation coverage (Miao et al., 2016). It can be inferred that the vegetation coverage or pollen productivity near the core LHK has clearly increased over time, with a notable increase observed between 2001 and 2022. The pollen assemblage was dominated by herbaceous pollen, followed by woody pollen, with few fern and algae spores. In pollen zones III and IV, the percentage of herbaceous pollen clearly increased, while the percentage of woody pollen, fern, and algae spores clearly decreased, indicating that the plant communities in the Liaohe Estuary were dominated by herbaceous plants from 1944 to 2022, and the percentage of herbaceous plant increased clearly between 2001 and 2022. The findings of this study, that plant communities during this period were dominated by herbaceous species and the percentage of herbaceous pollen increased over time, are consistent with conclusions from other studies conducted in the Liaohe Estuary (Ma et al., 2016; Guo, 2019). The herbaceous pollen mainly included *Chenopodiaceae*, *Artemisia*, *Poaceae*, *Cyperaceae*, and *Typha*, while the woody pollen was dominated by *Pinus*, deciduous *Quercus*, and *Betula*. These results are consistent with pollen records from other studies in the Liaohe Estuary and its surrounding areas (Yang et al., 2021, 2022). Furthermore, the ratio of *Chenopodiaceae* to *Artemisia* can be used to determine the dry and wet conditions of the period. A ratio greater than four indicates that the climate of the region is relatively dry, while a ratio lower than four indicates that the climate is relatively humid. The larger the numerical gap, the more significant the dry and wet conditions (Wang et al., 2010). The entire sediment profile in the core showed that the percentage of *Chenopodiaceae* gradually increased with decreasing soil depth and was the dominant group starting from pollen zone II, whereas the percentage of *Artemisia* decreased with decreasing soil depth (**Figure 3**). The *Chenopodiaceae*/*Artemisia* ratio gradually increased from 0.74 to 3.45, accompanied by a decrease in the percentage of aquatic species’ pollen, indicating that the climate of the region was initially relatively humid but exhibited a drying trend over time (**Figure 2**). Woody pollen was mainly *Pinus* (37.91%–46.19%), followed by *Betula* and deciduous *Quercus*, all of which are cold-resistant plants (Wang et al., 1986; Zhang et al., 2020).

Furthermore, we analyzed the pollen community characteristics and climate change in the four pollen zones according to changes in the percentage of different families and genera. First period (1944—1966 a): Pollen concentration in the entire soil profile was the lowest during this period, with herbaceous pollen dominant, indicating that the pollen community mainly comprised herbaceous plants. Woody pollen included *Pinus*, *Betula*, deciduous *Quercus*, *Ulmus*, and *Salix*, with *Pinus* being the only conifer, but accounting for a large proportion. The *Chenopodiaceae*/*Artemisia* ratio was 0.74. Pollen of several aquatic species such as *Cyperaceae* and *Typha*, as well as moisture-loving fern spores such as *Pteris*, was recorded. Herbaceous pollen included cold-loving *Asteraceae*, and woody pollen included cold-resistant species such as *Betula*, indicating that the climate during this period was humid and cool (Wang et al., 1986; Singh and Pusalkar, 2020). Second period (1966—1986 a): Pollen concentration increased clearly during this period, and the pollen zone distribution was similar to that in the first period, indicating that the pollen community was still dominated by herbaceous plants. The percentage of pollen from aquatic species, such as *Typha* and *Cyperaceae*, decreased, while the percentage of *Chenopodiaceae* clearly increased, with a *Chenopodiaceae*/*Artemisia* ratio of 1.18, indicating that compared to the period of 1944–1966, the climate was slightly drier during this period, but overall still humid. The proportion of coniferous species decreased slightly, indicating that the temperature during this period was slightly higher than that that during the first period, and the distribution of tree families and genera indicated a cool and humid climate similar to that of the first period. Third period (1986—2001 a): In this period, the pollen community was still dominated by herbaceous plants. Among woody pollen, we observed pollen of cold-resistant tree species, such as *Betula* and *Juglans*, as well as deciduous broad-leaved tree species, such as deciduous *Quercus* and *Juglans*, indicating a cool climate (Wang et al., 1986; Zykin et al., 2017). Among herbaceous pollen, *Chenopodiaceae* continued to increase clearly compared to their abundance compared with second period, with a *Chenopodiaceae*/*Artemisia* ratio of 1.94, indicating that humidity during this period continued to decrease compared to the second period, and the climate was cool and humid. Fourth period (2001—2022 a): Pollen concentration clearly increased and the percentage of herbaceous pollen exceeded 90%, being the highest in the entire profile. Compared with the third period, the percentage of woody pollen clearly decreased, with coniferous species such as *Pinus*, broad-leaved species like *Betula* and deciduous *Quercus* present in small quantities. Fern and algal spores were also scarcely represented. The pollen community was dominated by herbaceous pollen, among which the percentage of *Chenopodiaceae* continued to rise compared to the third period, reaching the highest level in the entire profile, with a *Chenopodiaceae*/*Artemisia* ratio of 3.45. The overall climate was characterized as cool and humid, but drier than that between 1986 and 2001. Other study on the Liaohe Estuary also indicate that, influenced by climate change and human activities, the area of the typical Chenopodiaceae plant *S. salsa* significantly increased from 2009 to 2015 (Chen et al., 2018).

### 4.2 Driving factors of plant community changes in the Liaohe Estuary

Changes in the structure and composition of plant communities are consequences of changes in the number of the main plant communities (Song et al., 2024). To better understand the characteristics of plant communities in the Liaohe Estuary during different periods, PCA was used to analyze the most dominant seven pollen taxa. The first principal component accounted for 41.2% of the total explanatory power, among which *Chenopodiaceae* showed a negative loading pattern and other pollen taxa showed the opposite trend (**Figure 4**). Studies have shown that in estuaries, *Chenopodiaceae* pollen is a reliable indicator of salt marsh environments, and the trend of pollen percentage change is well correlated with soil salinity and pH (Liu et al., 2023). The opposite loading pattern of *Chenopodiaceae* pollen and other pollen in the PCA in this study may be related to changes in environmental factors, such as soil salinity and pH. In studies of plant communities using pollen analysis, pollen composition and concentration have been widely used as important indicators of sedimentary environments (Miao et al., 2016; Yang et al., 2021). Pollen concentrations indicate that changes in vegetation cover or pollen productivity are related to agricultural production; therefore, climate, soil, and human activity factors were chosen, combined with the percentages of main pollen taxa and pollen concentration, to determine the factors affecting the composition, vegetation cover and pollen productivity of plant communities in the Liaohe Estuary (Jin et al., 2020).

Changes in temperature, precipitation, and other climatic factors are the main forces driving the changes in plant community characteristics. Studies on pollen and plant communities usually use climatic factors, such as temperature and precipitation, to investigate the patterns of plant community changes (Li et al., 2014; Yang et al., 2019). Redundancy analysis (RDA) revealed that MAT (*p*< 0.001) influenced the percentages of the most dominant seven pollen taxa and pollen concentration, while the correlation between MAP and the most dominant seven pollen taxa as well as pollen concentration was relatively weaker (**Table 1**).Studies have shown that rising temperatures have driven an increase in annual total pollen emission, leading to changes in pollen productivity (Zhang and Steiner, 2022). Additionally, vegetation coverage across the country exhibits a significant positive correlation with temperature (Qi et al., 2020). The results revealed a positive correlation between MAT and pollen concentration, particularly during the period from 2001 to 2022, where the increasing trends in MAT and pollen concentration were consistent. This indicates that the rise in temperature over the past 80 years has promoted vegetation coverage or pollen productivity in the Liaohe Estuary.

Due to the unique geographical characteristics of estuaries, changes in MAP primarily affect the species composition of plant communities by affecting soil salinity, rather than directly driving plant community changes (Song et al., 2024; Huang et al., 2024). Additionally, estuarine plant communities exhibit strong adaptability to typical environmental pressures, such as salinity and tidal changes. Alongside MAP, other water sources, such as groundwater and tidal influx, may help maintain plant growth, making these communities less sensitive to changes in MAP (Guan et al., 2011; Gao et al., 2010; An et al., 2017). The results of this study further showed that MAP had no significant impact on the most dominant seven pollen taxa and pollen concentration, suggesting that MAP was not a primary driver of plant community changes in the Liaohe Estuary over the past 80 years.

According to the RDA and random forest results, soil salinity, pH, and grain size were significantly correlated with the most dominant seven pollen taxa (*p*< 0.001, **Figure 5**), and made significant contributions to PC1 of the most dominant seven pollen taxa (**Figure 6**), which is consistent with findings from the Yellow River Delta (He et al., 2009; Ma et al., 2012). Studies in the San Francisco Estuary have shown that during periods of high soil salinity in estuary, the percentages of *Chenopodiaceae* and *Poaceae* pollen are higher, whereas the percentage of *Cyperaceae* pollen is lower (Byrne et al., 2001). In this study, soil salinity and grain size were clearly positively correlated with the most dominant seven pollen taxa, with a stronger correlation observed for *Chenopodiaceae* pollen. Between 2001 and 2022, the percentage of *Chenopodiaceae* pollen increased clearly, while the percentage of aquatic pollen, such as *Cyperaceae* and *Typha*, decreased. This suggests that the main plant taxa in the Liaohe Estuary exhibit strong adaptability to changes in salinity and grain size. These findings align with previous research on plant communities in the Liaohe Estuary (Dong et al., 1995). Research in the Mu Us Sandy Land has shown that vegetation cover is significantly negatively correlated with soil grain size and significantly positively correlated with soil pH (Chen et al., 2022). The RDA results in this study also revealed that soil salinity and grain size were negatively correlated with pollen concentration, while pH was positively correlated with pollen concentration (**Figure 5**). Additionally, the random forest results showed that soil salinity and grain size had substantial contributions to pollen concentration (**Figure 6**). It indicates soil salinity and grain size in the Liaohe Estuary contributed to an increase in vegetation cover, whereas soil pH had a negative impact on vegetation cover.

Agricultural production leads to changes in land-use patterns that affect the species composition and community richness of plant communities, thereby influencing plant community characteristics. Previous studies have shown that a decrease in the percentage of native pollen and an increase in agricultural pollen in estuarine sediments during certain periods are linked to human activities such as land reclamation, agricultural planting, and vegetation burning (Deng et al., 2006; Yang et al., 2013; Abrahim et al., 2013). Additionally, chemical agents used in agriculture, such as herbicides, can reduce the distribution of native plants and lower the species composition and diversity of plant communities (Qi et al., 2020). The present study showed that agricultural production was significantly correlated with the percentages of the most dominant seven pollen taxa and pollen concentration (*p*< 0.001, **Table 1**) and has substantial contributions (**Figure 6**). Specifically, it showed a negative correlation with the most dominant seven pollen taxa, particularly *Artemisia* and *Poaceae*, indicating that agricultural activities over the past 80 years have negatively impacted the main plant taxa of the Liaohe Estuary plant community, with a stronger inhibitory effect on *Artemisia* and *Poaceae*. However, agricultural production was positively correlated with pollen concentration (**Figure 5**), suggesting that while it negatively impacted the main plant taxa, it contributed to an increase in local vegetation cover or pollen productivity. These findings align with research on the Yellow River Estuary (Jin et al., 2020).

The results indicate that rising temperatures have promoted increased vegetation cover or pollen productivity. Increases in soil salinity and grain size enhance the dominance of main plant taxa (e.g., *Artemisia* and *Poaceae*) but reduce local vegetation cover or pollen productivity. Although agricultural production inhibits the dominance of key plant taxa (e.g., *Artemisia* and *Poaceae*), it enhances local vegetation cover or pollen productivity. Therefore, it is recommended to optimize plant community structure by regulating soil salinity and grain size in estuarine management, while rationally planning agricultural activities to reduce negative impacts on native plants and maintain species diversity and ecological functions. Additionally, monitoring and adaptation strategies for climate change should be strengthened to promote the sustainable development of estuarine ecosystems.

## 5 Conclusion

This study employed ^210^Pb dating, pollen analysis, combined with soil physicochemical properties such as pH, salinity, and grain size, as well as environmental factors including MAT, MAP, and agricultural production, to investigate the characteristics of plant communities in the Liaohe Estuary from 1944 to 2022 along with the key environmental factors driving these changes. The findings of this study provide a scientific basis for the vegetation restoration and management of the degraded ecosystem in the Liaohe Estuary. The results revealed that herbaceous plants dominated the plant communities in the Liaohe Estuary during this period, with a cool and humid climate that gradually became drier over time. Over the time span from 2001 to 2022, accompanied by the increase in MAT, the percentage of herbaceous plants in plant communities and the pollen concentration both exhibited the characteristic of increasing as the soil depth decreased; correspondingly, the percentages of trees, ferns, and algae showed a decreasing trend as the soil depth decreased. Soil salinity, grain size, and agricultural production were identified as the primary factors affecting plant communities and pollen concentration in the Liaohe Estuary. Natural factors such as salinity and grain size supported the dominance of the main plant taxa but reduced overall pollen concentration, whereas human-driven agricultural activities inhibited the dominance of the main plant taxa while increasing the pollen concentration.

## Data Availability

The original contributions presented in the study are included in the article/**Supplementary Material**, further inquiries can be directed to the corresponding author/s.
