# Supplementary figures and images for: Long-term dynamics and driving mechanisms of plant communities in a temperate estuary in eastern China based on pollen analysis: a case study of the Liaohe Estuary
Source: Front Plant Sci. 2025 Apr 28;16:1578390. doi: 10.3389/fpls.2025.1578390 (PMC12066701; doi:10.3389/fpls.2025.1578390)

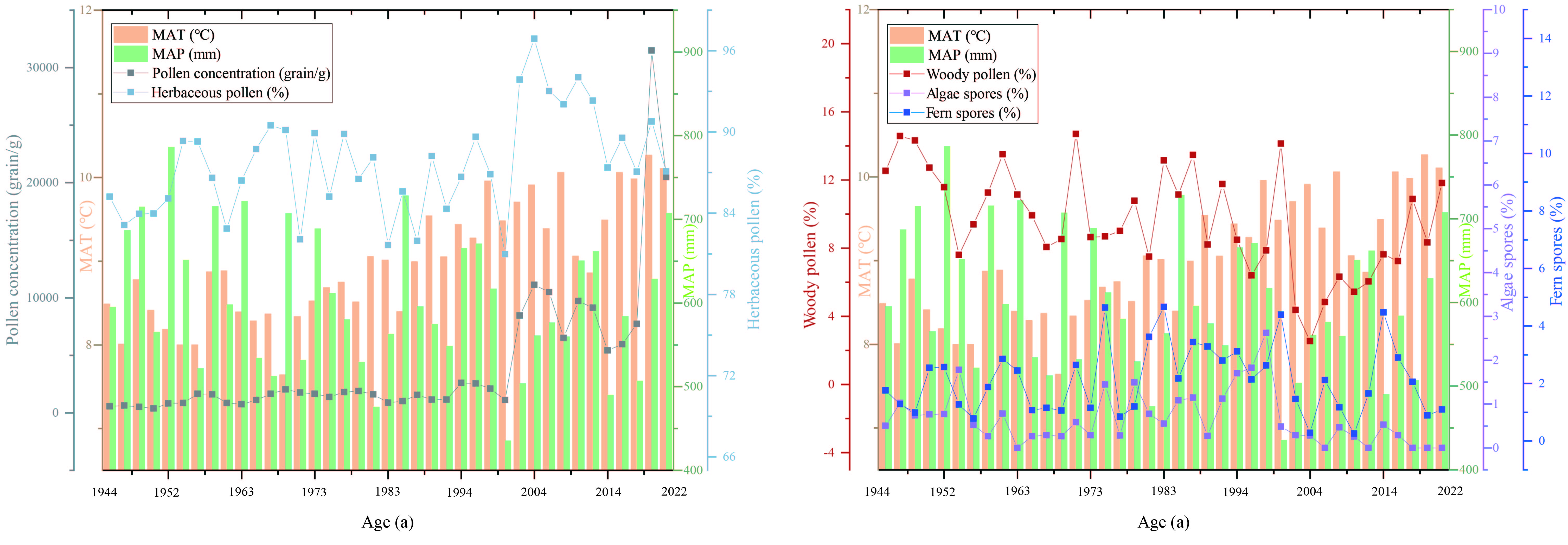

Supplement: Supplementary file 1 [file Image1.jpeg]
